# Treacherous Elasticity, Callous Boundaries: Aspiring Volunteer Initiatives in the Field of Refugee Support in Rotterdam

**DOI:** 10.1007/s11266-020-00260-3

**Published:** 2020-08-19

**Authors:** Lieke van der Veer

**Affiliations:** Department of Anthropology and Development Studies, Radboud University, Postbus 9104, 6500 HE Nijmegen, The Netherlands

**Keywords:** Refugees, Volunteering, Inequality, Active citizenship, Integration governance

## Abstract

This contribution focuses on volunteer initiatives that seek to assist refugee status holders in Rotterdam. It studies initiatives that are still in the process of fine-tuning their focus, grappling for funds, searching for volunteers, and seeking collaborations. The article lays bare the inequalities that such aspiring initiatives can be premised on and produce. In analyzing moments in which the label of ‘volunteer’ is rejected—or instead celebrated or transformed—this article demonstrates that the elastic representation of volunteering clashes with callous boundaries between ‘being only a volunteer’ and ‘doing something together.’ These boundaries are heartfelt by the organizers of these aspiring initiatives, who often have a refugee background themselves. By understanding inequality in volunteering in relation to debates about active citizenship, this article seeks to examine the workings of the glass ceiling that hinders the organizers of volunteer initiatives to transition into a position they consider more credible and professional.

## Introduction

In the Netherlands from 2015 onward, the ‘spectacle’ of people arriving to seek refuge was channeled by vast media attention and political debate. This so-called ‘refugee crisis’ (Casas-Cortes et al. 2015; De Genova 2018)—laying bare political upheaval, chaos, experimentation, inability, and unwillingness—triggered a vast response of bottom-up volunteer initiatives wanting to assist these newcomers. Based on a 12-month ethnographic fieldwork period in Rotterdam, this article highlights the inequalities that such initiatives can be premised on and produce.

This article proposes a refinement to analytic and empirical accounts that adopt a universalistic perspective to volunteering while excluding certain actors and activities from the realm of volunteering through processes of purification (Shachar et al. 2019). It suggests to understand such exclusions in relation to debates about active citizenship. This theoretical lens is relevant because, like universalistic perspectives to volunteering, the appeal to active citizenship is critiqued for summoning static publics and negative others and is intertwined with contemporary shifts in society that the initiatives studied are affected by. Specifically, concerning both constructed phenomena (volunteering and active citizenship), it is noted that vulnerable and especially the newest members of the population (including recently arrived refugees), in particular, face the risk of being cast in a disadvantageous position (Schinkel and Van Houdt 2010; Tomlinson 2010). In analyzing ethnographic material that presents practices of volunteering in the light of the active citizenship agenda, the article aims to foster a relational understanding of the hierarchies and inequalities that sustain and exacerbate social boundaries (Lamont and Molnár 2002; Alba 2005).

With reference to the apparent elasticity of volunteering, this article shows that, in the context of refugee support, volunteer activities shapeshift across different contexts and embody a range of modalities. In terms of modalities, this article demonstrates that volunteering may function as a way to respond to the appeal to active citizenship, as well as a way to self-organize in order to reclaim one’s own voice. It may also be an act to ‘give something back’ and to undo unequal power relations between giving and receiving. Despite its elasticity, however, this article demonstrates that the representation of volunteering as universal (Shachar et al. 2019, 257) clashes with sociologically complex boundaries (Alba 2005) that are heartfelt by community organizers who are in the process of starting a volunteer initiative in the field of refugee support and often have a refugee background themselves. This article demonstrates how the elasticity of volunteering treacherously invisibilizes these boundaries and shows how volunteering may function as a situation of subordination in which one may feel entrapped.

Rotterdam is an illuminating case to study volunteer initiatives in the field of refugee support. The city self-identifies a ‘policy laboratory,’ cherishes its alleged hands-on mentality—captured by the popular slogan ‘actions speak louder than words’—and has the highest number of low-income households in the Netherlands. Against this backdrop, however, Livable Rotterdam [‘Leefbaar Rotterdam’], currently the largest political party in the city, is a right-wing party with populist traits that particularly profiles around immigrant assimilation and is explicitly anti-immigration—which translates into policy frameworks that the volunteer initiatives that are studied are affected by. I began this fieldwork about 2 years after the building of the reception center for asylum seekers in Rotterdam led to violent protests (RTV Rijnmond 2015). In response to these protests, a platform that hosts refugee support initiatives was founded.

## Literature Review

### Refugee Support Initiatives

Long before 2015, third-sector organizations took on engagement with migrant integration (Garkisch et al. 2017). What’s new in the current situation, however, is the public perception on the mediatized transnational refugee movement in combination with the ‘overall perception of state failure in organizing refugee protection in Europe’ and the ‘organized non-responsibility’ of almost all EU member states (Pries 2019, 6; cf. Larruina et al. 2019, 54). In this context, the recent upsurge of refugee support initiatives are considered to include ‘new volunteers’ (Fleischmann and Steinhilper 2017) and constitute forms of ‘new volunteerism’ (Mayer 2018). These new volunteers and forms of volunteering are characterized as ‘spontaneous volunteering’ (Boersma et al. 2018; Pries 2019) or ‘humanitarian volunteering’ (Theodossopoulos 2016; Youkhana and Sutter 2017). By seeking to engage in ad hoc ‘crisis management’ regarding refugee reception (Boersma et al. 2018; Larruina et al. 2019), these individuals and initiatives compensated for the national governments’ orchestrated inertia. Although some initiatives vanished after the initial phase of immediate care and assistance, others ‘created structures out of the first spontaneous practices’ (Hamann and Karakayali 2016), and ‘trends of professionalizing’ were observed (Pries 2019, 2).

Whereas some of these studies seem to stage ‘volunteers’ as somehow preexisting and homogeneous entities, other studies acknowledge the multiplicity of forms of volunteering that responded to the ‘spectacle’ of people seeking refuge since 2015 and draw attention to the indeterminateness of their position. For instance, Mayer (2018) highlights heterogeneity within the field volunteering by sketching various ‘dispositions’ to provide support to refugees (237) and Fleischmann and Steinhilper (2017) exposed some of the ‘ambivalent and, at times, contradictory effects’ (24) of volunteering. Larruina and Ghorashi (2016) highlighted the ‘duality of the insider–outsider position’ that volunteers in a Dutch reception facility are faced with. Rast and Ghorashi (2018) found that, ‘given the unpaid character of volunteer work, many newcomers perceived the status, value, and benefits of volunteering as quite limited’ (Rast and Ghorashi 2018, 193). The authors point out that, although refugees demonstrate ‘active participation as volunteers’—a state of affairs that can potentially ‘deepen democracy’ (189)—the respondents in their study attributed a ‘low status and limited benefits… to volunteer work’ (193).

### Active Citizenship

The massive amount of people ‘registering as volunteers’ for ‘traditional volunteer organizations’ (Rast et al. 2019, 2), as well as the proliferation of emerging volunteer initiatives, can in part be contextualized with the move to an activating welfare state in the Netherlands. This trend can be traced back to the political discourse from the 1970s onward (Hajer 2011; Tonkens 2008; Verhoeven 2009), and led to the appraisal of ‘voluntary organizations’ as ‘the most direct expression of citizens’ commitment’ (Larruina et al. 2019, 56). For example, the Ministry of the Interior and Kingdom Relations (2013) declares that they ‘have faith in social initiatives’ that want to ‘help active citizens’ by for example ‘inviting non-active citizens and [calling for] good volunteer policies.’

Shachar et al. (2019) perceive of this process, in which governments encourage citizens to be volunteers, as part of ‘hybridization’ of volunteering. The active citizenship agenda proposes a normative judgment concerning what citizenship should entail. This agenda is related to mechanisms of responsibilization (Schinkel and Van Houdt 2010), which makes citizens, the private sector, or communities responsible for public tasks previously considered the sole responsibility of the state (van Houdt and Schinkel 2019). As a consequence of this active citizenship agenda and responsibilization mechanisms, the figure of the volunteer appears highly mediated—a state of affairs that opened up the possibility to study the process in which volunteers are ‘crafted’ (cf. Rozakou 2016).

Paradoxically, the image of self-entrepreneurial and active citizens emphasizes individual responsibility and at the same time conceives of the citizen as acting in line with the government’s objectives (Dean 1999; Muehlebach 2007). As will be shown, I found that these unfulfilled promises of self-entrepreneurialism appear at the heart of refugee status holders’ dissatisfactions in trying to start their own initiative. Although the dichotomy of paid employment and unpaid labor analytically oversimplifies the complexity of work practices (Taylor 2004), being offered a volunteer position—as opposed to getting help to become a ‘self-entrepreneur’—feels like being turned down. When understanding refugee status holders’ attitude toward unpaid work vis-à-vis paid work in relation to their economic and social position (Taylor 2005, 135), what becomes clear is that unpaid work does not fit with their images of professionalism and successfully settling in—images mediated by the appeal to active citizenship.

### Repressive Responsibilization and Social Boundaries

In addition, for refugee status holders, their efforts to volunteer and self-organize are not only embedded in the appeal for active citizenship in which they have to be ‘active’ by doing volunteer work and initiating community projects. It is observed that, especially in Rotterdam, the image of the active citizen produces ‘its negative other: the passive, immoral, irresponsible, inactive citizen targeted by repressive (correctional or disciplinary) interventions’ (van Houdt and Schinkel 2019). The authors refer to this mechanism as ‘repressive responsibilization’ (Schinkel and Van Houdt 2010) which labels those who are not seen as not fully ‘integrated’ in the ‘moral community’ (Schinkel 2017) as ‘potential threat to social order’ (van Houdt and Schinkel 2019, 709)—including people who are unemployed, people who are illiterate, and people with an immigrant background.

In Rotterdam, such repressive responsibilization is materialized, for example, in the ‘Participation Act,’ a ‘workfare’ measure (Krinsky 2007; cf. Kampen 2014). Although this measure holds for everybody who receives benefits—and is motivated by the idea that volunteering would lead to paid work—for refugee status holders, it is called the ‘Time Obligation’ [dagdeleneis]. This measure obliges refugee status holders who receive benefits to do volunteer work—yet perceiving of programs such as the ‘Time Obligation’ as ‘volunteering’ is ‘obviously highly questionable’ (Shachar et al. 2019). The policy document ‘The Rotterdam Approach to Status Holders: 2016–2020’ points out that ‘Rotterdam has the ambition that status holders are active in society for at least 4 days a week or more with education, work, or voluntary work’ (Rotterdam City Council 2015, 16–17).

The appeal to active citizenship and the repressive responsibilization thus presuppose social boundaries (Lamont and Molnár 2002; Alba 2005), in the sense that incorporation into the moral community is made conditional upon desired forms of participation. People with a refugee background are expected to do volunteer work in return for benefits and demonstrate the self-entrepreneurship that underpins active citizenship—hurdles that must be jumped in order to be admitted as deserving community member. However, what will be shown is that, for refugee status holders, in attempting to flow from volunteering into starting a volunteer organization and to ultimately live up to the ideal of moral citizenship, a glass ceiling is met with.

### Shifting Positions in the Gift Exchange

Alongside the appeal to active citizenship (that applies to all residents) and repressive responsibilization (that applies to status holders as well as, for example, people who are unemployed), for status holders specifically, there are additional motivating factors to do volunteering. Rast and Ghorashi (2018) point out that, for refugee status holders, motivations to volunteer include learning the language, developing a network, making friends, becoming part of Dutch society, working with computers, getting to spend time outside, and changing negative discourses regarding refugees (192). Moreover, the authors found that, for some of the refugee status holders in their study, ‘voluntary engagement’ was an ‘exchange’ for lessons that were taken, and that, via volunteering, one could ‘give back to the Dutch’ and thereby ‘feel more equal’ (Rast and Ghorashi 2018, 192).

‘Giving back’ to the community more commonly is referred to as an aspect of volunteering (Krinsky and Simonet 2017, 190–194). Regarding ‘immigrants and refugees’ specifically, Weng and Lee (2016) note that ‘these populations’ constitute ‘rich resources for volunteerism and giving back,’ especially with respect to ‘voluntary activities contributed to one’s own ethnic community’ (511). Vandevoordt (2017), in his study on the practices of homeliness by Syrian refugees in Belgium, applies the notion of ‘giving’ to granting hospitality to autochthone Belgians. The author noted that ‘giving’ as opposed to receiving shifts his interlocutors’ position in the gift exchange, relieves them of their status as dependent, and as such reverses the power relations between hosts and guests. Implicit in these accounts is the ontology in which volunteers are cast as debtors (Krinsky and Simonet 2017, 190–194), and that, for refugees, the debt is cast as more absolute—for their claim to ownership of territory and community is not grounded in participation that precedes this debt (Schinkel and Van Reekum 2019).

Little research, however, exists about refugee status holders that themselves launch a volunteer organization in the field of refugee support. Boersma et al. (2018) note that ‘many volunteers who started or joined bottom-up initiatives had refugee backgrounds’ and that, by virtue of engaging in such initiatives, these volunteers demonstrated that they are ‘agents of change’ (731). However, as will be shown, I contend that joining or starting an initiative should analytically not be folded into one another, for this would obscure the glass ceiling between these two activities. Mayer (2018) mentions the importance of ‘self-organized refugee movements’ (241) in Europe that make visible grievances through resistance (cf. Nyers and Rygiel 2012; Ataç, Rygiel and Stierl 2016; Baban and Rygiel 2017; Stierl 2019), and Tsavdaroglou (2019), in his study on a self-organized initiative by refugees residing in the Idomeni settlement in Greece, shows that the will to challenge official media representations of refugees and to reclaim the voices of refugees at the camp were an incentive to start ‘an initiative.’ Yet, as will be shown, putting-up resistance and the demonstrating a will to challenge media representation as explanatory factors do not exhaust my interlocutors motivations to start an initiative; for a large part, they just want to be seen as credible and professional.

## Methods and Scope

This article is based on a 12-month ethnographic research in Rotterdam between January 2018 and December 2018. By studying initiatives that are still fine-tuning their focus, grappling for funds, searching for volunteers, and seeking collaborations with others et cetera, I gained insight in the constitutive and generative elements of the infrastructure of refugee support and reception (cf. Harvey et al. 2017). Moments of breakdown (Larkin 2013) were particularly insightful; when my research participants hoped for or anticipated something that did not arrive (Reeves 2017), I learned about who may do what, where and how.

The initiatives I studied seek to assist people who have received a refugee status after 2015 and who have been relocated to Rotterdam to start their new life there. What the initiatives share is a motivation to relieve discomfort, unsafety and precarity (Dijstelbloem and Van der Veer 2019) to people who were forced to flee their former homes. Moreover, the initiatives studied are not established yet, but are still in the process of becoming. These factors functioned as criteria for the purposive sampling of people, settings, and situations. Upon entering the field, I found out that, in Rotterdam, volunteer initiatives were often initiated by residents with a refugee background themselves. Although the ethnographic examples in this article are mainly suchlike initiatives, the scope of my fieldwork is not limited to ‘migrant self organizations’ (Van Heelsum 2004; Rijkschroef and Duyvendak 2004; Uitermark et al. 2005).

Initiatives were mainly selected at an event that took place three weeks into fieldwork. At the ‘market of initiatives,’ this event showcased initiatives that provide support to refugees. At that market—a collection of tables covered with brochures and attended by people who run the respective initiatives—I arranged follow-up meetings that brought about participation in the research. Typically, I followed aspiring initiatives in their efforts to find partnerships with other organizations, joined them to meeting sessions—e.g., with people who give advice on funding circuits, with civil servants to discuss their project proposals—and participated in events they organized that were aimed at both refugees and volunteers. In doing so, via snowball sampling, contact with some nongovernmental organizations as well as activities related to local politics was established. In addition, I contacted other nongovernmental organizations—often upon recommendation by existing research participants. I owe to a number of gatekeepers that functioned as key participants and provided me with permission to carry out my research—their various positionalities pointing to the fragmentation of authorities vis-à-vis forms of access (Rozakou 2019). Regarding complex methodological decision-making regarding ethical and epistemological issues in politicized circumstances (Maillet et al. 2017), I always considered the welfare of my interlocutors—who I consider extraordinarily courageous in responding to the challenges of contemporary life (cf. Chapkis 2010, 495).

I followed six aspiring initiatives throughout their start-up phase and conducted numerous open interviews with the organizers of these initiatives. I joined them to 13 meeting sessions with people who proposed to help them in professionalizing their initiative. Also, I participated in ten meetings between initiatives as well as brainstorm meetings within initiatives and joined 16 events aimed at refugee status holders impact sessions for sponsors. I conducted 12 open interviews with policymakers, heads of funding organizations, and civil servants such as ‘area networkers’ and ‘area connectors.’ Also, I conducted six additional open interviews with employees who work at nongovernmental organizations that are contracted by the municipality, managers of community centers, and employees who work at the local reception center. With reference to local politics, I visited ten meetings of ‘management committees,’ ‘area committees,’ and local election-related profiling events and protests. In order to understand the bureaucratic classifications and procedures (Wedel et al. 2005; Riles 2006) regarding integration and self-organization, I collected local and national policy documents. Lastly, I collected internal reports, written summaries of meetings, e-mail threads, and flyers.

Anthropologists, in studying life as it is experienced, are part of the world that they study, and their findings are intersubjective and situational (Chapkis 2010; Small 2009). In negotiating my position as researcher, making explicit previous engagement in the field of refugee support pushed my research forward—for this prevented my endeavors from being experienced as ‘crisis chasing’ (Cabot 2019). At the same time, in line with Bagelman (2016), I felt ‘at once drawn to and disturbed by various modes of […] support that I have witnessed and participated in’ (Bagelman 2016, 8). The ambivalence of these contradictory and complex experiences—that were as well reflected in the experiences of my interlocutors themselves—guided my research (cf. Chapkis 2010, 489). The ways in which these experiences limit or enable ethnographic work cannot be solved via a strictly individual response (Chapkis 2010, 486). However, interlocutors sometimes joked that I was also a ‘some kind of newcomer’—given that I moved to Rotterdam for fieldwork. Simultaneously, interlocutors sometimes made fun of the fact that, in their experience, my profile met with that of a typical NGO worker—given that I was seen as ‘a woman from Dutch heritage asking questions.’ Outside of such bantering, my interlocutors valued that the fact that I spent considerable time to establish close relationships that were grounded in empathy and carefully attended to their experiences.

I kept a detailed research diary—sometimes in a notebook, sometimes on my phone, sometimes directly on my laptop—that included conversations, observations, and reflections, which I typed in every evening after fieldwork. The analysis involved several phases of sorting and labeling the ethnographic material—in accordance with thematic and descriptive categories that emerged in the iterative-inductive process of identifying patterns. For instance, for this article, I largely drew on material labeled as ‘pursuing collaborations’ and ‘volunteer appreciation’—issues that were often brought up explicitly by the interlocutors. I analyzed the interlocutors’ experiences in relation to the broader forces that shape these experiences and pursued an extended case method by tracing how certain key events linked (Burawoy et al. 1991; Mitchell 1983; Small 2009). I use pseudonyms for all the names of my research participants as well as the volunteer initiatives in order to protect their identity. My identity as a researcher was made known to all parties involved. Participation was secured (and often reconfirmed) by informed consent, after having disclosed information about the research project, the benefits of the study, and procedures regarding confidentiality.

## Findings

This section brings into dialogue the appeal to active citizenship with the proliferation of volunteer initiatives in Rotterdam that seek to assist refugees. It shows that, while some forms of volunteering are celebrated, not every volunteer or every volunteer initiative is applauded with equal enthusiasm. By attending to moments in which research participants feel kept down as ‘just’ volunteer and analyzing moments in which the label ‘volunteer’ is rejected—or instead embraced or transformed, this section demonstrates inequalities between different volunteers and forms of volunteering.

### Differential Appreciation

On one night in April, I was invited to a ‘residents’ night’ that was organized by a large welfare organization that is contracted by the municipality, together with the local ‘area committee.’ An area committee is an elected board of residents (who are not necessarily on the list of a political party but often are) that bring out advice to the city council and are involved with managing the budget that is available for volunteer initiatives. Approximately, 200 people composing the audience—a collection of residents, civil servants, and people working for welfare organizations—sit around a large theater stage in a recently rebuilt neighborhood center. The purpose of the night, the ‘area chair’ [gebiedsvoorzitter] announces just after the lights that shone on the spectators are dimmed, is to ‘pamper the volunteers.’ In his speech to commence the program, he exclaims that ‘There are a lot of people who want to contribute!’ and that ‘Politics has never been so close to the citizen!’ Then, the actual host of the night, a woman wearing a colorful dress and braided flowers through her hair, is invited to award residents a pin for their engagement in the neighborhood. The volunteers that are awarded include: someone who organizes a sports tournament, someone who does ‘activities with mothers,’ and someone ‘who is HIV positive but knew to recuperate in a positive way.’ Throughout the ceremony, the host attempts to stimulate the audience and cheers into the microphone: ‘We are so happy that people are so active!’ and ‘This is how it goes in the participation society [participatiemaatschappij]!’.

I sit next to Bob, an 85-year-old man who has lived in the neighborhood all of his life was a neighborhood worker [buurtwerker] for long and is greeted with respect by the people who walk by his chair. He is not impressed by what is happening on the stage. With reference to the celebration of volunteer initiatives, he says: ‘This is all not as noble as it seems. Because if Moroccans want something, then they get nothing. And these people [pointing at the stage], they do not see that. This is Rotterdam. ‘Livable’ [‘Leefbaar Rotterdam,’ currently the largest political party] wanted to lower the flag to half-mast [to emphasize mourning] when Aboutaleb [member of the Dutch Labor Party PvdA who is from Moroccan decent] was elected as a mayor.’ And even Jasper, who runs the neighborhood center, is skeptical. When I later ask him how he feels about the celebration, he says, about the volunteers: ‘Is it work, or is it volunteering? These people get 250 euros from the area committee. They’re entrepreneurs. It’s a good way to put bread on the table [je brood verdienen].’

What I get from Bob’s and Jasper’s commentaries is that there are implicit selection mechanisms for which volunteer initiatives are invited to this kind of celebration. Bob suggests that the influential right-wing party would, in one way or the other, prevent ‘Moroccans’ from entering the realm of successful participation. This differential inclusion remains out of sight at this gleeful celebration of volunteers. Mayer (2018) too noticed that, regarding volunteering in the field of refugee support, ‘the symbolic appreciation of volunteers,’ who are given ‘honours and prizes at reward ceremonies’ proliferates (240–241). In addition, what this ceremony lays bare is that some activities are considered as ‘active’—such as volunteering in the neighborhood—but others not (Van den Berg 2016).

### Abundant Initiatives and the Appeal to Self-Organization

‘This whole fuzz with everything organized for refugees; I sometimes wonder whether it is actually good for them’—said a project assistant of an NGO that helps status holders to me. With ‘fuzz,’ she refers to the proliferation of outings, consultations, dinner groups, trainings, festivities, and buddy programs for people with a refugee background that are allocated to Rotterdam and need to find their way in setting up their new lives. She is by far not the only practitioner that, throughout my fieldwork, expressed concerns about the abundance of initiatives that responded to the mediatized spectacle of the influx of refugees. Aida, a woman who is born in an East African country, fled to the Netherlands and started a foundation that offers administrative help to (mainly Eritrean) refugees, stated that ‘people are just tired of refugee initiatives. There are enough initiatives.’ In one of the many visits to her office, we sat on the pavement together drinking coffee. She says: ‘Refugees are so hot. It’s a big topic. There are so many foundations. Also foundations that were not even bothered with refugees before are now making some kind of project. It’s big business.’

During fieldwork, I came across several people who had recently been given a refugee status themselves—mainly young men in their twenties that fled from the war in Syria and sought refuge in the Netherlands—who were motivated to start an initiative to care for (other) former refugees. Tawfik, who fled to the Netherlands in 2014 and now gives several trainings for refugee status holders, some time said: ‘In 2014 I was helped by Dutch people, the Central Agency for the Reception of Asylum Seekers [COA], the Refugee Council. So now I am a little bit old in the Netherlands. I know a lot. And if Dutch people help [refugees], I have to help twice as much. I experience this as a duty.’

Sahir, who had also recently fled from Syria and who now volunteers in distributing a local newspaper, helps around in a community center, organizes neighborhood festivals, and fixes people’s clothes, has a similar disposition toward his activities. During an information night and Iftar dinner (daily fast-breaking meal during Ramadan) organized by the foundation ‘Hands On,’ Sahir was invited to the stage to introduce himself. He grabbed the microphone without hesitance and said: ‘I am Sahir. I do volunteer work. And 3 days a week, I learn the language. And I fix people’s clothes. I really like people in the Netherlands.’ He goes on, and says, with fire: ‘I like my neighbors [buurman en buurvrouw]. I like to have contact with people. Voluntarily.’ Sahir continues with his pitch: ‘A lot of people. All the time. As a volunteer. Every day.’ Later that night, I mention to Sahir that I was taken by his kind words. He responds with urgency: ‘I have to say that! I do it for other people! I don’t forget that these people try to help me! So I know that I have to also help them. When I say nice things about them, people will come to them. And that’s also good for me. So that is why I say that I do things voluntarily!’

### ‘Doing Something Back’ and ‘Reclaiming One’s Voice’

Zahed is a young man in his twenties who fled Syria a few years ago and who is making plans to start ‘an initiative,’ together with his friend Nizar. Once, when I joined Zahed to a cafe where he would pitch ‘his story’ to an aspiring author who wants to write a book about Zahed, he said to the author: ‘We have arrived in a community that welcomed us, so we want to do something back.’ Some other time, again in a cafe, together with a colleague of mine, Zahed said to my colleague: ‘I need to demonstrate how Syrian people suffer. People should listen to refugees themselves.’ It is telling that this is what he is saying to people who are not me—the author, my colleague. Because to me, he has a slightly different story, a gloomier one.

One night, when I was sitting with Zahed on his rooftop, he said: ‘So yeah, we need to write a plan, and we need to say that we are going to help the communities in Rotterdam’—his voice being theatrical, and bored out at the same time, underlining the repetitiveness of the vocabulary that he so much seems to recognize as a policy rhetoric. He continues, again demonstrating that he is aware of whatever ‘works’ in this scene, with the same skeptical tone: ‘So we are, of course, going to work with volunteers, and we will work with refugees, to help to give them a future, help them with jobs, integration, bla bla bla.’ On the one hand, Zahed thus mobilizes the motivational narratives of ‘giving something back to the host society’ and of ‘giving a voice to the refugees.’ On the other hand, Zahed is deliberately using the vocabulary of ‘active citizenship’: he speaks to this appeal by crafting a positive story about volunteers and communities, demonstrating his understanding that this is expected of him in order to settle in and be seen as a good, responsible and entrepreneurial citizen.

A similar thing holds for Jamal. Like Zahed, Jamal recently fled from Syria and thinks of ‘starting an initiative.’ But over dinner in a Syrian bar, he once said that sometimes he just ‘pretends’ he wants to do so—because he figured out that talking about his aspirations to ‘start an initiative’ would give him some credibility, some legitimate reason to have something to chat about to others, to have a plan. Both Jamal and Zahed deploy a terminology that echoes descriptions of the subjectivity of the ideal citizen and juggle with policy jargon with the hope of being recognized as professional. However, as it turns out, both Jamal and Zahed feel that, in trying to pursue their own goals, the contracted nongovernmental organizations that said to help them through the start-up phase of their initiative do not allow them to move beyond the category of volunteer. As such, the value of self-entrepreneurship that the appeal to active citizenship seemed to promise for them, would not be redeemed—as the ethnographic material below shows.

### ‘Being Only a Volunteer’ Versus ‘Doing Something Together’

Just a few months into fieldwork, when I talked with Zahed and his friend Nizar about their plans, I asked whether they happened to have turned to an organization named ‘Roots.’ ‘Roots’ is contracted by municipality. Based on an interview I had with its founder earlier that year, I thought that, for Nizar and Zahed, ‘Roots’ could maybe take Nizar and Zahed on board. Nizar, however, says, factually: ‘I have talked with the founder. For five min some time. He said he liked our idea. But he wants us to work as volunteers, whereas we want to do something together. Not only be a volunteer.’ I nodded and asked whether they have talked to ‘Refugees Up,’ another contracted nongovernmental organization. Nizar said: ‘They are also part of, like, the official. For these people, it is about numbers. They get money.’

To Nizar and Zahed, being offered a volunteer position feels like simply being palmed off. They feel discouraged by the seeming impenetrability of glass ceiling between ‘being only a volunteer’ and ‘doing something together.’ ‘Being only a volunteer’ does not fit with their images of professionalism and successfully settling in. The ‘fantasy of employability’ (Bloom 2013) that they sustain thus clashes with their interactions with nongovernmental organizations—by which they feel turned down.

I often had similar conversations—like the one with Nizar and Zahed—in which research participants denounced being cast ‘only’ as volunteer. And quite some times, I saw firsthand how such casting works, especially in meetings where refugee status holders who dream of launching their own volunteer initiative seek collaborations with people in the field that proposed to help them. Jamal, for example, who heard that the aforementioned contracted nongovernmental organization ‘Refugees Up’ runs the project ‘Victoria,’ to help status holders start an initiative, made an appointment with this organization early 2018. Jamal’s idea was to organize a summer school for status holders to prepare them for studying at a university. The founders of ‘Refugees Up’ were positive, and they decided to get two young women who are both long-term residents on board as well. These young women joined the meetings on how to organize this summer school, together with Jamal, an employee who works for ‘Refugees Up,’ and me.

During these conversations, it soon became clear that Jamal was mainly turned to when discussing how to get participants for the summer school—assuming Jamal would introduce ‘Refugees Up’ to status holders he is expected to know. At the same time, the two young women are mostly concerned with what their ‘salary’ would be in co-organizing the summer school. At one meeting, when the two women were negotiating their ‘salary’ with the employee who works for ‘Refugees Up,’ Jamal asks, a bit shy: ‘So how does that work actually?’—after he had been silent for quite a long time. The employee who works for ‘Refugees Up’ says: ‘Good point’ and asks to Jamal, rhetorically: ‘Maybe you can get a volunteer compensation?’ Jamal looks a bit puzzled and does not respond. The conversation continues. Later, when the two young women talk about ‘compensation for the hours they make for workshop development,’ Jamal asks: ‘How do you mean, workshop development?’ The employee from ‘Refugees Up’ then asks: ‘Do you ask this because you want to also develop workshops?’ Jamal, politely, says: ‘Maybe,’ although it all had been his project plan from the start. The employee then seems to realize this too and says: ‘Yes, maybe this is good to keep in mind. Because it’s your idea after all, Jamal. And now the money goes to you, ladies. […] With pay rolling, Jamal could maybe get paid as well. Then he doesn’t need to have a volunteer compensation. Think about it, Jamal.’

Jamal never got paid. In the following meetings, the topic of compensation was not brought up again, and Jamal was too shy to do so himself. The meetings went by, discussing the details of the summer school—including what to put in the goody bags and where to print the flyers. In August, one of the young women suddenly proposed to Jamal, as if it were a generous gesture: ‘Maybe you can get a volunteer compensation?’—as if nothing of the previous conversations took place. In the end, the negotiations did not reach a critical point; the summer school was canceled because the municipality declined their approval for funding.

## Discussion and Conclusion

This article discussed how the appeal to active citizenship—which prescribes that in order to be seen as a moral and responsible citizen one should be engaged with the community—mediates the willingness to volunteer in the field of refugee support. The conditionality that this ideal of active citizenship presupposes is based on an inequality between those who are indeed ‘active’ and those who are not. For people with a refugee background who do volunteer work or are (in the process of) launching their own (volunteer) initiative, this appeal manifests itself even bleaker. In embarking on a journey that would lead to moral citizenship, it appears that they are behind from the start. Despite the motivation to do something back as well as the motivation to reclaim one’s voice, the role that is available to the former refugee who is keen on launching a volunteer initiative sometimes is ‘only’ that of a volunteer. The qualification ‘only’ is in place here because the aspiration was exactly to move beyond the role of volunteer. The glass ceiling of not ‘just’ being a volunteer and instead to overcome one’s vulnerable position through volunteering and to become truly self-entrepreneurial is not easily broken.

Much as the performance of a vocabulary of active citizenship—and its emphasis on making a positive contribution to the community—is manifest in the ways in which Zahed, Jamal and Nizar position themselves, the ultimate promise of the successful self-entrepreneur is yet out of reach. They feel that they are not taken seriously by the contracted organizations that are supposed to help aspiring initiatives; because the success of these organizations is in part measured by the quantity of volunteers they attract, the aspiring community organizers experience that they are used instrumentally for other people’s key performance indicators, kept down as volunteer, and as such are trapped in an unequal position. The apparent elasticity of volunteering thus is gashed by callous boundaries that are felt as an obstacle to settling and living up to the ideal of moral citizenship.

Overlapping materializations of inequality became visible in reviewing the ways in which some volunteers and forms of volunteering are not recognized, supported, and celebrated. These materializations of inequality include repressive assumptions that underlie images of moral citizenship—that manifest themselves in the ways that former refugees who try to launch a (volunteer) initiative are confronted with a hard-boiled burden to prove their eligibility. And they include subjectivities of volunteering in which volunteers are instrumental to larger organizations’ impact factors—that were disclosed when these former refugees, in their efforts to professionalize their aspirations, were kept down in their role as volunteer, in the face of their yearnings to be seen as more than that. Further research is needed in order to investigate the nature of these interlinked forms of inequality, as well as the conditions under which aspiring community organizers with a refugee background are treated differentially.
